# Translating and Adapting the Modified Mini Mental State (3MS) Test for Multiple Cultural Populations

**DOI:** 10.1093/geroni/igab046.3536

**Published:** 2021-12-17

**Authors:** Tara Rose, Evelyn Teng, Helena Chui, Katherine Erickson, Chia Ying Chen, Elyse Manzo

**Affiliations:** 1 University of Southern California, Los Angeles, California, United States; 2 University of Southern California, Moraga, California, United States; 3 University of Southern California, Huntington Beach, California, United States

## Abstract

Within healthcare settings, screening of cognitive abilities in older adults is routinely conducted for the detection, early intervention, and management of cognitive impairments. The Modified Mini Mental State (3MS) test takes approximately 10 minutes to administer and has a score range of 0 - 100. It can provide an estimated MMSE score, and has been used in multiple countries since 1987 with approximately 1,900 publications. The United States has many diverse populations with different languages and cultural backgrounds. How to appropriately translate and adapt the original 3MS test in English for each minority group in order to better serve them is an important issue. Cross-cultural assessment involves much more than accurate translation of test items across languages. One needs to know not only the oral and written languages involved, but also the life experiences and circumstances of the target populations. This presentation first covers some general considerations in test translation and adaptation, including attention to cultural, ecological, and language specifics. We shall then present Chinese, Korean, and Hindi 3MS record forms to illustrate the reasons and ways for modification of some of the test items. To accommodate different writing systems, for example, 3MS test versions with an alphabet are different from ones with logographic character representations. Modifications of the 3MS items include those on temporal orientation, spatial orientation, naming, and repetition. In summary, cultural, ecological, and linguistic differences must be taken into account for cognitive screening in order to enhance cross-population comparability and be more inclusive for aging ethnic minorities.

